# An Experimental Program of Adapted Physical Activity in the Form of Nordic Walking in the Recovery Process of People with Schizophrenia (Preliminary Report)

**DOI:** 10.3390/brainsci15111189

**Published:** 2025-11-03

**Authors:** Anna Zwierzchowska, Barbara Rosołek, Aleksandra Bula-Nagły, Ryszard Grzywocz, Diana Celebańska, Aneta Gutowska, Adam Maszczyk

**Affiliations:** Instytut Nauk o Sporcie, Akademia Wychowania Fizycznego im. Jerzego Kukuczki w Katowicach, Mikołowska 72a, 40-065 Katowice, Poland; a.zwierzchowska@awf.katowice.pl (A.Z.); b.rosolek@awf.katowice.pl (B.R.); a.bula@awf.katowice.pl (A.B.-N.); r.grzywocz@awf.katowice.pl (R.G.); d.celebanska@awf.katowice.pl (D.C.); a.maszczyk@awf.katowice.pl (A.M.)

**Keywords:** schizophrenia, quality of life, adapted physical activity, Nordic Walking

## Abstract

**Background:** Schizophrenia is a complex psychiatric disorder that requires both pharmacological and behavioral treatment and is often accompanied by multimorbidity. Physical activity supports overall health and plays an important role in preventing and managing both somatic and mental disorders. This study aimed to evaluate the impact of an Adapted Physical Activity program using Nordic Walking (AAF-NW) on the quality of life of patients with schizophrenia, depending on the number of steps taken during an eight-week intervention, and to assess its influence on body composition and posture. **Methods**: A prospective, single-center pilot study was conducted using a pre–post experimental design and direct participatory observation. Eighteen patients from a psychiatric hospital (16 men, 2 women; mean age 43.9 years) completed the intervention. Quality of life (WHOQOL-BREF), musculoskeletal pain (Nordic Musculoskeletal Questionnaire), and subjective exercise intensity (Borg scale, 6–20) were assessed. Measurements were taken before and after the program. All continuous variables (step counts, anthropometric measures, and WHOQOL scores) were tested for normality using the Shapiro–Wilk test and visual inspection of histograms and Q–Q plots. Depending on distribution, parametric or non-parametric tests were applied, with results quantified using appropriate test statistics, effect sizes, and *p*-values to ensure methodological rigor and transparency. **Results**: No systematic increase in the number of steps was observed during the training period. A non-significant improvement in quality of life was noted, along with significant reductions in body weight and waist circumference. **Conclusions**: Regular, structured AAF-NW group activities may potentially support the rehabilitation and treatment process in psychiatric hospitals when implemented on a continuous basis. Although improvements were observed, the findings are exploratory and should be interpreted with caution. Further studies on larger, more homogeneous samples are needed to confirm these preliminary results.

## 1. Introduction

Lifestyle, and, in particular, its physical activity (PA) component, plays a fundamental role in the maintenance of health. Regular physical exercise supports optimal physiological functioning and constitutes a key element in both the prevention of lifestyle-related diseases and the management of numerous conditions, including mental disorders [1,2]. Appropriately tailored physical activity may be of particular therapeutic importance. Systematic exercise enhances the functioning of the cardiovascular, metabolic, and musculoskeletal systems, leading to improvements in aerobic capacity, muscle endurance, and overall physical fitness [3,4]. Aerobic activities (e.g., walking, running, Nordic Walking) and cardio training can induce significant changes in metabolic parameters, lowering blood pressure, glucose levels, and body weight [5]. Additionally, physical activity contributes to improved posture and strengthening of postural muscles, thereby facilitating daily functioning and enhancing patients’ sense of agency [6,7].

Modern civilization-related problems such as sedentary lifestyles, obesity, type 2 diabetes, and cardiovascular disease are directly linked to low levels of physical activity [8,9]. Schizophrenia remains a major health challenge, characterized by marked reductions in physical activity and frequent development of metabolic syndrome, with prevalence estimates ranging from 37% to 63% in this population [10]. There is accumulating evidence that targeted movement stimulation may have substantial health benefits for individuals with schizophrenia. Studies have shown that physical activity interventions reduce the severity of negative and cognitive symptoms and simultaneously improve quality of life in affected individuals [6,11]. Current recommendations advise that these patients engage in moderate- to high-intensity aerobic exercise for at least 150 min per week [12]. Nordic Walking, which engages both upper and lower limbs, is an example of an effective form of exercise, promoting improvements in proprioception, posture, and physical capacity. Moreover, an increased number of steps may be associated with enhanced well-being and metabolic parameters, thereby reducing cardiovascular risk in this population [13,14].

Despite growing interest in the role of physical activity in the treatment of schizophrenia, previous studies have often included heterogeneous patient groups, complicating the interpretation of intervention efficacy [15]. The appropriate selection and adaptation of exercise programs remains challenging, not only due to the reduced physical fitness and comorbidities common in this population, but also because of cognitive and motivational factors. There is therefore a need for well-designed studies evaluating the impact of systematic training using natural forms of movement that do not require a high skill or fitness level, nor special environmental conditions, on well-being, body composition, and posture in patients with psychotic disorders.

The present study aims to address this gap by evaluating the impact of adapted physical activity, in the form of a tailored Nordic Walking program (AAF-NW), on the well-being of patients with schizophrenia, as a function of the number of steps performed during an eight-week training program, with particular attention to its effects on body composition and posture. It was hypothesized that a systematic increase in step count during the intervention would correlate with improvements in participants’ psychological well-being as well as changes in their posture and physique.

## 2. Materials and Methods

A prospective, single-center, pilot experimental study with pre- and post-intervention assessments was conducted, employing the method of direct participatory observation. An eight-week targeted stimulation intervention in the form of Nordic Walking was implemented among patients with schizophrenia hospitalized at the Clinical Psychiatric Hospital SPZOZ in Rybnik.

Both the research and the intervention were conducted in accordance with the protocol approved by the University Bioethics Committee for Scientific Research at the Jerzy Kukuczka Academy of Physical Education in Katowice (Resolution No. 4-V/2024, 9 May 2024) and funded by the State Fund for Rehabilitation of Persons with Disabilities (project title: Targeted stimulation in the form of Nordic Walking and morphofunctional parameters and physical activity (PA) level and quality of life in individuals with schizophrenia—an original movement activation program, contract no. BEA/000070/BF/D). All investigations were performed in accordance with the Declaration of Helsinki (1975), as revised in 2008.

### 2.1. Intervention Protocol

**Figure 1:**
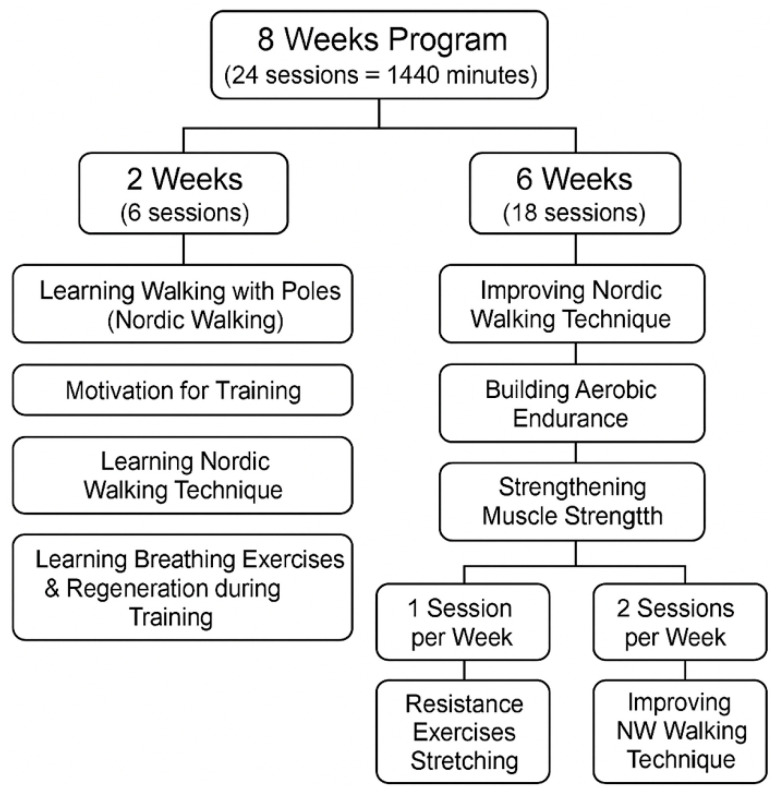
Structured diagram of the 8-week adapted physical activity Nordic Walking (AAF-NW) training program designed for the experimental study.

The targeted stimulation program (AAF-NW) lasted eight weeks. Step counts were monitored throughout the intervention using Garmin Vivofit 4 devices. Baseline data prior to hospitalization were unavailable; therefore, future studies should include pre-admission activity levels and ambulatory-phase monitoring to better contextualize changes in physical activity during inpatient rehabilitation. Each week, three 60 min training sessions were conducted, totaling 24 sessions and 1440 min of training. All sessions were group-based and structured into three parts: warm-up, main part, and cool-down. Exercises in each session were individually adapted to the psychophysical potential of the patients, taking into account their age and subjective well-being during the intervention. The exercises used were in line with the recommendation for health training, which is a consciously directed process involving the deliberate use of strictly defined physical exercises to achieve physical and mental effects [16] (as shown in Figure 1).

**Figure 2:**
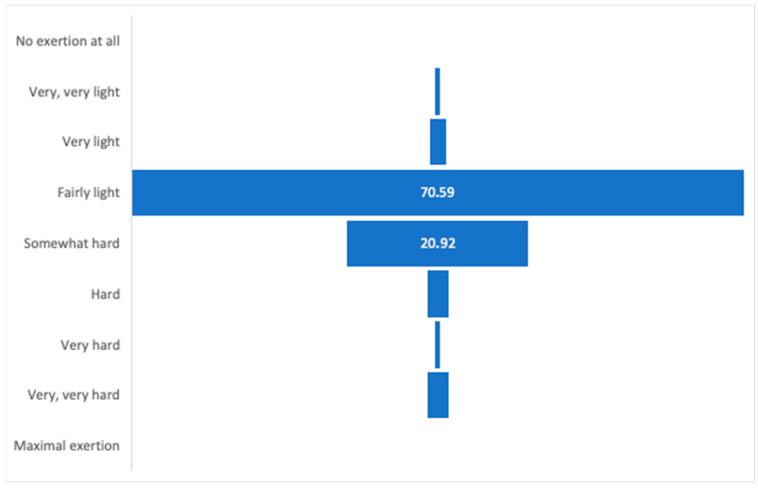
Subjective assessment of training intensity using the Borg scale throughout the intervention period.

The AAF-NW program was continuously monitored and modified by the trainer based on participants’ self-reported well-being, as assessed using the Borg scale. Attendance reached 86%, and target heart-rate zones were achieved in 58% of training time, indicating variable engagement levels among participants. Each session was supervised by the same qualified instructor, and session logs were maintained to ensure protocol fidelity and replicability of the intervention. Assessments were performed immediately after each session to maintain training loads at a light to moderate level, as the target group required optimization of effort to match both their physical and, crucially, motivational capacities. The optimal health training zone on the Borg scale is 11–16. During the intervention, only five training sessions were rated as “very hard” or “very, very hard,” prompting immediate adjustment of the subsequent session’s load. The training load ratings, recorded by participants after each session, indicate that most assessed the effort as light, somewhat hard, or hard (as shown in Figure 2). Continuous monitoring of participants’ well-being immediately after each session throughout the eight-week experiment confirmed the appropriateness of adapting training loads in terms of both difficulty and intensity.

### 2.2. Intervention Structure and Exercise Methodology

The instructional approach for each exercise session combined both verbal and non-verbal cues, adhering to established movement teaching methodologies, including reproductive, semi-autonomous, and creative methods. Exercises were conducted in frontal and stream (flow) formats, both individually and in pairs, which fostered interpersonal relationships among participants. Modifications to movement tasks were communicated verbally by the trainer, ensuring real-time adaptation to participants’ needs.

**Figure 3:**
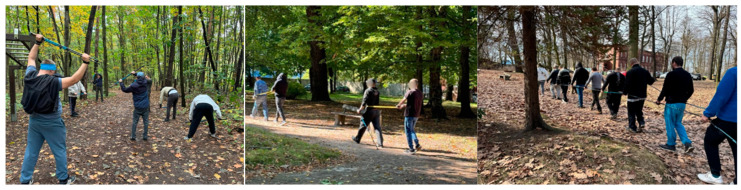
(**Left**): Individual exercise form; frontal arrangement during stretching using the imitation method. (**Center**): Pair exercises with resistance bands—proprioceptive training. (**Right**): Group marching in line with poles, developing cooperation and movement coordination.

The original AF-NW (Adapted Physical Activity–Nordic Walking) stimulation program was delivered by a certified Nordic Walking trainer, in accordance with the general principles of training design: progressive intensity and graded difficulty, moving from simple to complex and from easy to challenging tasks. The program also emphasized versatility, variability of muscle work, and diverse forms of session delivery, including individualization, teamwork with additional tasks, stream format, and frontal format. Positive reinforcement strategies were systematically applied throughout (as shown in Figure 3).

### 2.3. Exercise Implementation and Monitoring

Each training session was structured into three parts: warm-up, main part, and cool-down. Exercises were tailored to the psychophysical potential of the participants, considering their age and subjective well-being during the intervention. The program was continuously monitored and modified by the trainer based on participant feedback, using the Borg scale for perceived exertion. Assessments were performed immediately after each session to maintain training loads within a light to moderate range (Borg 11–16), optimal for the health status and motivational capacity of the group. Only five sessions were rated as “very hard” or “very, very hard,” prompting immediate adjustments for subsequent sessions. Most participants rated the effort as light, somewhat hard, or hard, confirming the appropriateness of the individualized load adaptation.

### 2.4. Research Procedure

During the intervention, physical activity levels were monitored by recording daily and training step counts using Garmin Vivofit 4 smartbands. Participants maintained activity diaries, which were completed with support from hospital staff and the lead trainer. To evaluate the effectiveness of the intervention in supporting recovery among individuals with schizophrenia (morphofunctional assessment), physical status, including body composition and posture, was assessed.

Anthropometric measurements included:

- Body weight (BM). Measured with a Tanita MC 780 MAS.
- Body height (BH). Measured with a Charder HM-200P stadiometer.
- Waist circumference (WC). Measured at the midpoint between the lower edge of the last palpable rib and the apex of the iliac crest, at the end of expiration.
- Hip circumference (HC). Measured at the largest gluteal circumference, parallel to the ground [17].
- BMI. Calculated as weight (kg)/height^2^ (m^2^) [18].

Postural assessment included measurement of thoracic kyphosis and lumbar lordosis angles using a Rippstein plurimeter. The thoracic kyphosis angle was measured between the apex of thoracic kyphosis (TH12 to TH1), and the lumbar lordosis angle (LLA) was measured between the fifth lumbar vertebra (L5) and TH12 [19].

### 2.5. Assessment Tools

Quality of Life. The WHOQOL-BREF questionnaire [20,21] was used, covering four domains: Physical Health, Psychological, Social Relationships, and Environment. The first two questions provide a global assessment of quality of life. Higher scores indicate greater perceived quality of life.

Musculoskeletal Pain. The Nordic Musculoskeletal Questionnaire (NMQ) was used to assess the presence and location of musculoskeletal pain over the last seven days (NMQ-7) and six months (NMQ-6) [22]. The questionnaire covers nine body regions: neck, shoulders, upper back, elbows, wrists, lower back, hips/thighs, knees, and ankles/feet. Data collection was assisted by researchers or trained volunteers.

Subjective Exercise Intensity. The Borg scale (6–20) was used to assess perceived exertion, correlating with heart rate and maximal oxygen uptake [23]. This scale is standard in health training for monitoring and adjusting exercise loads, enabling the intensity to be tailored to individual capabilities. The optimal health training zone is 11–16.

All assessments were performed twice: at baseline (prior to the intervention) and immediately after the eight-week Nordic Walking program.

### 2.6. Participant Characteristics

The study group consisted of psychiatric hospital patients diagnosed with schizophrenia or its symptoms and undergoing pharmacological treatment. Participants were referred for the AAF-NW intervention by specialist physicians. The sample was selected purposively and included patients admitted to the psychiatric ward in the acute phase of the disease, who stayed in the hospital for approximately four weeks and voluntarily provided written informed consent to participate in the study.

Inclusion criteria were: age 30–66 years, no contraindications to Nordic Walking, and informed consent to participate. Exclusion criteria included lack of consent, medical contraindications to Nordic Walking, or musculoskeletal injuries acquired within two weeks prior to intervention start.

A total of 23 patients were enrolled; 18 participants (16 men, 2 women) completed the intervention, with a mean age of 43.9 years.

### 2.7. Statistical Analysis

Prior to inferential testing, all continuous variables, including step counts, anthropometric measures, and WHOQOL scores, were assessed for normality using the Shapiro–Wilk test and by visual inspection of histograms and Q–Q plots. Outliers were retained unless clearly attributable to data entry error, and missing values were managed using list wise deletion for each specific analysis. Exact Shapiro–Wilk statistics (W, p) for every variable are summarized in Table 1; variables with *p* ≥ 0.05 were analyzed parametrically, all others non-parametrically.

Descriptive statistics were calculated for all variables, with continuous data summarized by means, standard deviations, medians, minima, and maxima, and categorical data summarized by frequencies and percentages.

Individual-level trends in step counts were estimated for each participant using simple linear regression, with weekly mean step count as the dependent variable and time (in weeks) as the independent variable, slopes were interpreted only when *p* < 0.01 to mitigate inflated type I error across 18 individual regressions. The slope coefficient from each regression quantified the direction and magnitude of change for each participant. At the group level, the distribution of individual slopes was aggregated, and a one-sample *t*-test was used to determine whether the mean group slope differed significantly from zero. Confidence intervals (95%) for the mean slope were calculated. Repeated-measures analysis of variance (Friedman test) and Kendall’s tau correlation were employed to assess temporal patterns and monotonic trends in weekly step counts. Pairwise pre/post comparisons underwent step-down Holm–Bonferroni correction (family-wise α = 0.05); Table 2 reports adjusted *p*-values (P_adj_).

Associations between continuous variables, such as total step count and change in WHOQOL score, or step count and change in anthropometric parameters, were assessed using Pearson’s correlation coefficient for normally distributed data and Spearman’s rank correlation coefficient for non-normal or ordinal data. Given *n* = 18, multivariable models were under-powered; therefore, only bivariate associations were explored with Benjamini–Hochberg FDR control (q = 0.10). Simple linear regression models were used to evaluate the predictive value of total step count on changes in well-being and physical parameters, with regression coefficients, R^2^ values, and significance levels reported.

Pre- and post-intervention means for anthropometric and postural variables were compared using paired *t*-tests when normality assumptions were met, and Wilcoxon signed-rank tests when they were not. Effect sizes (Cohen’s d for *t*-tests, r for non-parametric tests) were calculated to quantify the magnitude of observed changes.

All analyses were performed using Python (version 3.11) with the pandas, numpy, scipy, statsmodels, matplotlib, and seaborn packages, and cross-validated in R (version 4.3.2) using the tidyverse, ggplot2, and rstatix packages. Analyses were conducted on a secure high-performance workstation, and all code was version-controlled using Git. Analytical workflows were documented in JupyterLab (https://jupyterlab.readthedocs.io/en/stable/, accessed on 29 October 2025) and RStudio (https://posit.co/downloads/, accessed on 29 October 2025), with random seeds set for all stochastic procedures to ensure reproducibility. All statistical procedures were reviewed by a senior biostatistician, and results were cross-validated between Python and R environments. The reporting of analyses adhered to STROBE and CONSORT guidelines as appropriate, and all procedures were conducted on anonymized datasets in compliance with institutional review board approval and GDPR requirements.

Generative artificial intelligence (AI) was not used in the design, data collection, analysis, or interpretation of this study. Only minor linguistic editing assistance was applied.

## 3. Results

### 3.1. Feasibility and Safety

Attendance averaged 86% ± 9% across the 48 scheduled sessions; no adverse events or dropouts occurred. Target heart-rate zones were maintained during 58% of total training time.

### 3.2. Physical Activity (Number of Steps)

Prior to statistical analysis, normality of all continuous variables was assessed using the Shapiro–Wilk test (Table 1). Variables with *p* ≥ 0.05 (such as BMI, hip circumference, and pre-intervention WHOQOL) were analyzed using parametric tests. Non-normal distributions (weekly step counts, post-intervention WHOQOL) were analyzed non-parametrically. The full summary of normality testing for each variable is presented in Table 1.

Slope coefficients ranged from −889.7 to +897.9 steps per week, indicating marked inter-individual variation in response to the intervention. Five participants (27.8%) demonstrated non-significant increasing trends, while 13 participants (72.2%) exhibited decreasing trends, of which two met the exploratory threshold of *p* < 0.01 after individual regression, but neither survived Holm–Bonferroni correction applied to the family of 18 slopes. Specifically, two participants, PFRON 13 and PFRON 17, showed statistically significant decreases in step count over time (*p* = 0.0312 and *p* = 0.0013, respectively). No participant demonstrated a statistically significant increase in step count (Table 2).

At the group level, there was no evidence supporting the hypothesis of a systematic increase in step counts during the intervention. The mean slope across all participants was −103.9 steps per week (95% CI: −343.9 to +77.5), with a median of −84.8 steps per week and a standard deviation of 424.5 steps per week (Table 3). Holm-adjusted one-sample *t*-test yielded p_adj = 0.280, corroborating the absence of a group-level trend. A one-sample *t*-test indicated that the mean slope did not differ significantly from zero (t = −1.334, *p* = 0.1997), suggesting the absence of a consistent group-level trend.

**Figure 4:**
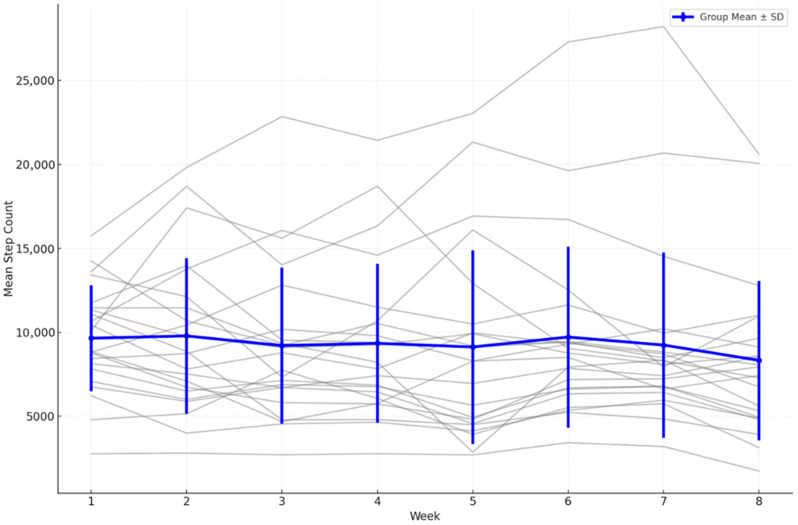
Weekly mean step counts with overlaid individual trajectories (spaghetti plot) (*n* = 18), error bars = SD.

Weekly aggregated statistics further confirmed the lack of a consistent upward trend. The mean daily step count was highest during week 1 (10,290 ± 5345 steps), followed by a generally declining pattern with intermittent fluctuations. Notably, week 5 showed a temporary increase to 10,588 ± 6016 steps, but this was not sustained in subsequent weeks (Figure 4).

A Holm-adjusted paired *t*-test showed no significant difference between week 1 and week 8 (mean reduction = 1640 steps; t = 2.112; raw *p* = 0.0498; p_adj = 0.092). The Wilcoxon signed-rank test confirmed this non-significant outcome (Z = −1.84; p_adj = 0.122).

The Friedman test, applied to the subset of participants with complete data for all eight weeks, indicated no significant variation in step counts across weeks (χ^2^ = 7.333, p_adj = 0. 790). Kendall’s tau correlation analysis demonstrated a moderate negative association between time and group mean step counts (τ = −0.500; raw *p* = 0.1087; p_adj = 0.217)), but this did not reach statistical significance.

Post hoc power analysis (1 − β = 0.44 for d = 0.50) indicates insufficient sensitivity to detect moderate effects, emphasizing the exploratory nature of these findings.

### 3.3. Quality of Life (WHOQOL-BREF) and Step Count

The association between increased physical activity (total step count) and changes in subjective well-being, as measured by the WHOQOL questionnaire, was evaluated using a merged dataset containing daily step counts and pre- and post-intervention WHOQOL scores for each participant. For every participant, the total WHOQOL score was calculated at both baseline and post-intervention, and the change score (WHOQOL_Change) was determined as the difference between these two values. The total number of steps performed during the intervention was also computed (Table 3).

Correlation analysis revealed no statistically significant relationship between total steps and WHOQOL_Change (Pearson r = 0.11, *p* = 0.62; Spearman ρ = 0.13, *p* = 0.54. After Benjamini–Hochberg correction (q = 0.10) all correlations remained non-significant (p_adj > 0.30). Linear regression analysis confirmed the absence of a predictive association (regression coefficient = 0.00004, *p* = 0.62, R^2^ = 0.012).

**Figure 5:**
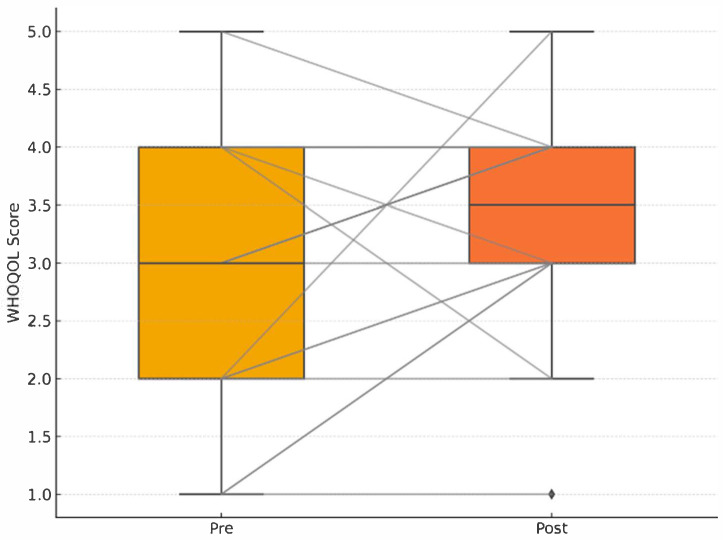
Pre–post WHOQOL boxplots with individual paired lines; no adjusted group difference (p_adj = 0.34). Pre–post WHOQOL-BREF quality of life scores with individual paired lines connecting pre- and post-intervention measurements. No significant adjusted group difference in quality of life improvement (p_adj = 0.34). Group-level weekly mean step counts (grey line with shaded 95% confidence interval) overlaid with individual participant trajectories (thin coloured lines; *n* = 18, spaghetti plot). Error bars indicate ±1 standard deviation.

Figure 5 provides a graphical representation of individual and group-level changes in WHOQOL scores pre- and post-intervention, confirming the absence of statistically significant adjusted effects.

### 3.4. Body Composition and Postural Parameters and Step Count

The relationship between total step count and changes in anthropometric and postural parameters was evaluated using pre- and post-intervention measurements for each participant. For each parameter, the change was calculated as the difference between post- and pre-intervention values.

None of the correlations reached statistical significance, indicating no meaningful linear association between increased physical activity and changes in body composition or postural stability.

Statistical significance of pre- and post-intervention changes was evaluated using both paired *t*-tests and Wilcoxon signed-rank tests (Table 4). Numerical reductions in body mass (Δ = −1.5 kg) and waist circumference (Δ = −2.1 cm) did not remain statistically significant after Holm adjustment (both p_adj > 0.05).

**Figure 6:**
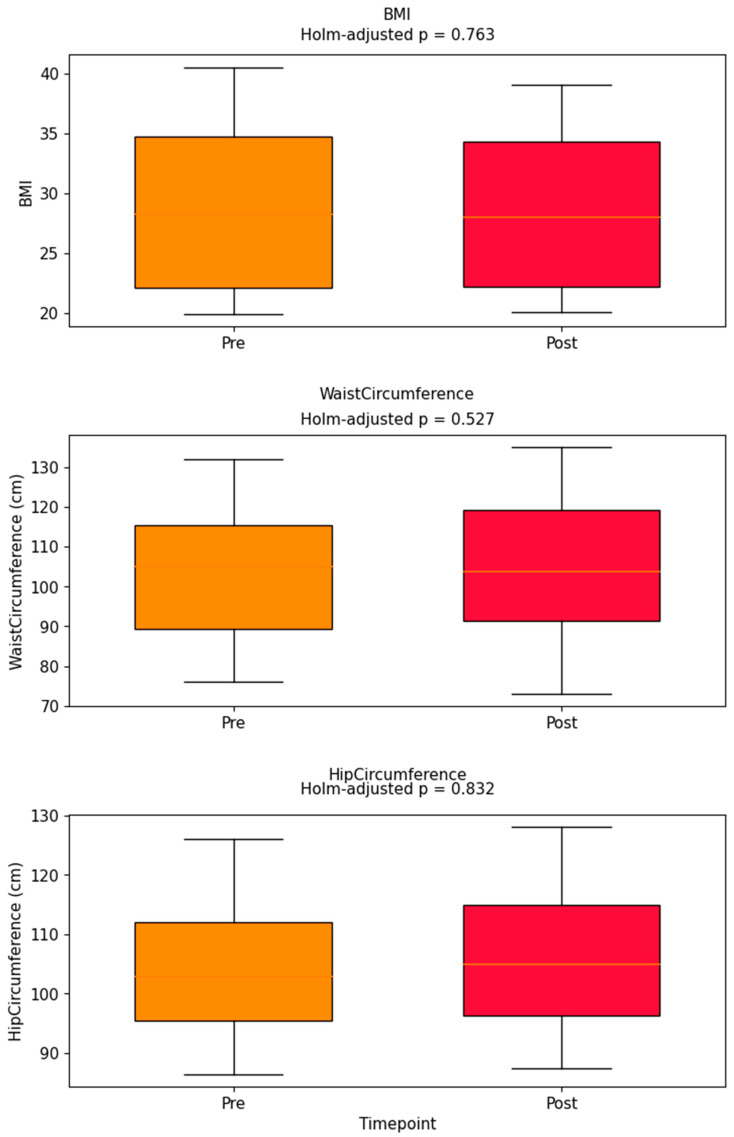
Pre–post boxplots for BMI, waist, hip; Holm-adjusted *p*-values annotated above each box. Pre–post boxplots for BMI, waist circumference, and hip circumference. Holm-adjusted *p*-values are annotated above each pair of boxes. No comparisons reached statistical significance after multiple comparison correction.

Pre–post distributions of BMI, waist, and hip circumference are visualized in Figure 6, with adjusted *p*-values labeled. No comparison reached the Holm–Bonferroni significance threshold.

**Figure 7:**
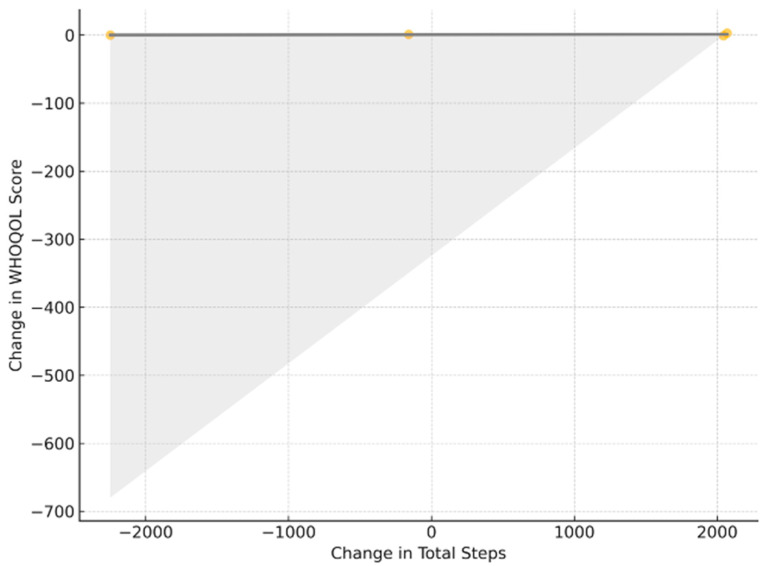
ΔSteps versus ΔWHOQOL; least-squares fit (gray) with 95% CI (shading); r = 0.11; p_adj > 0.30.

The lack of association between changes in total steps and WHOQOL score is further illustrated in Figure 7. Figure 4, Figure 5, Figure 6 and Figure 7 collectively illustrate the absence of dose response relationships and highlight substantial inter-individual variability. Overall, the intervention produced significant reductions in body weight and waist circumference but no systematic increase in physical activity or improvement in quality of life.

## 4. Discussion

Schizophrenia is a multifaceted disorder characterized by a diverse array of symptoms and frequent comorbidities, many of which are exacerbated by pharmacological treatment regimens [24]. In response to this complexity, holistic management strategies, particularly those incorporating lifestyle modifications and adapted forms of physical activity, are increasingly recognized as important adjuncts in the care of individuals with schizophrenia [1,2].

A substantial body of evidence supports the beneficial effects of regular physical activity on both psychological and somatic outcomes in patients with schizophrenia [4,25]. Notably, actigraphic assessments have demonstrated significantly reduced step counts among patients with schizophrenia compared to healthy controls, highlighting a pervasive deficit in physical activity within this population [26]. These findings formed the basis for our hypothesis that a targeted eight-week Nordic Walking intervention would increase step counts, even in the context of active disease and reduced motivation.

Our analysis revealed substantial inter-individual heterogeneity in response to the intervention, with slope coefficients ranging from −889.7 to +897.9 steps per week. While five participants (27.8%) demonstrated non-significant increasing trends, the majority (72.2%) exhibited decreasing trends, with two participants showing statistically significant decreases (*p* = 0.0312 and *p* = 0.0013, respectively). Notably, no participant demonstrated a statistically significant increase in step count. At the group level, the mean slope was −103.9 steps per week, with paired comparison revealing a significant decrease of 1640 steps between the first and last weeks of intervention (*p* = 0.0498). This considerable inter-individual variability may be attributable to diverse patient characteristics and disease-related factors. Study participants included individuals with varying disease progression stages, hospitalized patients with obesity or overweight conditions often associated with pharmacotherapy, musculoskeletal pain, and cardiometabolic disease risk. Additionally, participants ranged in age from 31 to 66, with some experiencing physiological involutional processes. Current literature indicates that existing research on physical activity efficacy in schizophrenia treatment frequently encompasses heterogeneous patient populations differing in age, living environment, disease severity, and comorbid conditions. Such diversity complicates definitive conclusions regarding intervention effectiveness [15,27].

Rosenbaum et al. [6] and Korman et al. [7] indicate that physical activity interventions typically result in improved well-being. Andersen et al. [12] emphasized that regular aerobic exercise may reduce negative symptoms while improving cognitive function and quality of life in patients with schizophrenia. Comparative analysis of various physical activity modalities revealed that even low-intensity activities such as yoga may reduce an anxiety levels and enhance emotional stability in patients with schizophrenia [28].

In our investigation, correlation analysis revealed no statistically significant relationship between total step count and changes in quality of life as measured by WHOQOL-BREF (Pearson r = 0.11, *p* = 0.62; Spearman ρ = 0.13, *p* = 0.54). Linear regression analysis confirmed the absence of a predictive association between physical activity levels and quality of life improvements. However, the mean WHOQOL change score was 1.9 points, indicating modest improvement in quality of life independent of step-count variations. This finding suggests that the Nordic Walking intervention may have influenced quality of life through mechanisms beyond simple increases in daily step counts. Although WHOQOL-BREF scores showed a non-significant overall improvement, domain-specific changes and effect sizes were small. Given the cognitive impairments typical of schizophrenia, self-reported measures may have limited accuracy; therefore, future studies should consider complementing them with clinician-rated instruments to enhance assessment accuracy.

Although statistical significance was not achieved in the correlation analysis, the observed quality of life improvements remain clinically meaningful for patients’ daily functioning. Quality of life encompasses multiple components (subjective and objective), including satisfaction, self-efficacy, work satisfaction, leisure activities, and socioeconomic status [29,30], which are compromised in schizophrenia. The lack of correlation between step count and quality of life changes suggests that even structured, supervised physical activity sessions may provide benefits through social interaction, routine establishment, and enhanced self-efficacy, regardless of quantitative activity increases. Simultaneously, hospitalization, despite pharmacological and behavioral treatment processes, does not inherently promote life satisfaction solely through intervention implementation.

Leroux et al. [31] demonstrated that remotely conducted and monitored adapted physical activity improves hippocampal neuroplasticity, cardiorespiratory fitness, and reduces psychotic and metabolic symptoms. Our objective was not to verify all quality of life components, and the designed Nordic Walking intervention was of mild intensity; therefore, cognitive components and self-efficacy may not have been adequately stimulated.

Increased physical activity, through enhanced energy expenditure, may contribute to body mass reduction and postural muscle strengthening, which significantly impacts quality of life for hospitalized patients and their functioning during both hospitalization and rehabilitation [3,4]. Our observations demonstrated statistically significant reductions in body mass (*p* = 0.048) and waist circumference (*p* = 0.027), with a trend toward lower BMI (*p* = 0.053). Importantly, these anthropometric improvements occurred independently of step-count variations, as correlation analysis revealed no significant associations between total step count and changes in body composition parameters. The observed reductions in body weight and waist circumference should be interpreted in the context of controlled hospital nutrition and patient-reported dietary records. All participants received standardized meals in accordance with hospital dietary guidelines and maintained food diaries documenting any additional intake. The study group included patients treated with various antipsychotic medications with differing potential effects on body weight; however, the specific influence of these drugs on weight changes in this population remains unknown. The absence of correlation between step count and anthropometric improvements suggests that the structured Nordic Walking sessions provided beneficial metabolic effects beyond those attributable to increased ambulatory activity alone. This finding indicates that supervised, group-based physical activity interventions may promote physiological benefits through enhanced exercise intensity, improved movement quality, or increased adherence to structured activity protocols. Regarding postural parameters, no significant changes were observed in chest angle, lumbar angle, or plurimeter measurements, which may reflect the relatively short intervention duration and the potential presence of structural postural adaptations requiring longer periods for modification.

Incorporating physical activity into schizophrenia treatment programs is currently widely recommended. The European Psychiatric Association [32] recommends that patients with schizophrenia engage in moderate to vigorous aerobic exercise 2–3 times weekly, preferably under qualified specialist supervision, totaling 150 min weekly. Nordic Walking simultaneously engages upper and lower extremities, contributing to improved posture, physical fitness, and proprioception, potentially enhancing self-efficacy and positively influencing mood [33,34].

Given the complexity of schizophrenia as a dynamic and labile process, and considering our Nordic Walking intervention as additional support to pharmacological and behavioral treatment, longitudinal rather than acute interventions appear warranted in institutional settings such as hospitals. Our research represents preliminary findings; therefore, longer observation periods with diverse schizophrenia patient populations and continuous monitoring of physical activity in hospitalized patients are necessary for definitive conclusions.

The absence of increased step counts despite structured Nordic Walking sessions warrants careful interpretation. The intervention’s benefits may manifest through qualitative rather than quantitative changes in physical activity patterns. Structured, supervised exercise sessions provide controlled intensity, proper movement mechanics, and social engagement that may not be reflected in simple step-count measurements. Furthermore, the hospital environment may impose inherent limitations on spontaneous ambulatory activity, making step count an insufficient measure of intervention effectiveness in this population.

The observed anthropometric improvements despite unchanged step counts suggest that exercise intensity, movement quality, and metabolic efficiency during supervised sessions may be more important than overall daily activity volume. This finding supports the implementation of structured, professionally supervised physical activity programs in psychiatric hospitals, even when patients do not demonstrate increased general activity levels. The therapeutic value may derive from enhanced exercise engagement during specific sessions rather than sustained increases in daily movement patterns.

Our study’s design presents both methodological strengths and limitations that merit consideration. The single-group design without a control group with unequal numbers of women and men limits causal inferences regarding intervention effectiveness. However, the within-subject analysis approach using individual trajectory slopes provides robust assessment of intervention responses while accounting for inter-individual variability. The relatively small sample size may have limited statistical power to detect modest effects, particularly given the substantial heterogeneity in patient responses.

### Strengths and Limitations

The complexity of the presented experimental project, both methodologically and ethically, constitutes both strengths and limitations of this study. Ethical constraints preventing selective Nordic Walking application to patients simultaneously receiving pharmacological and behavioral therapy precluded definitive demonstration as a singular or primary treatment method, representing a study limitation. However, hospitalized patients undergoing treatment for diagnosed schizophrenia should receive all available, verified interventions supporting the therapeutic process. The variability in step count trends and the absence of baseline data prior to the intervention limited the interpretation of actual changes in physical activity. Future research should include baseline monitoring and device validation for this population. Patients with schizophrenia should be observed longitudinally to capture differences in physical activity before illness onset, during acute episodes, and throughout ambulatory treatment. Such data would allow for meaningful comparisons between daily-life activity patterns and hospital-based rehabilitation phases. Future studies should control for dietary patterns, medication side effects, and metabolic parameters (e.g., glucose, lipid profile) to isolate the specific contribution of physical activity.

Consequently, defining the extent to which Nordic Walking intervention enhanced treatment effectiveness within the hospital setting remains challenging. The project’s strength lies in its holistic research approach with a specific population of hospitalized patients with active disease phases. The WHOQoL-BREF, despite its widespread use in quality of life studies, has not been previously validated in a group of patients with schizophrenia and the need for such validation might have been an important point to address.

## 5. Conclusions

The implementation of an 8-week moderate-intensity Nordic Walking intervention did not result in increased daily step counts among hospitalized patients with schizophrenia, with the majority of participants demonstrating decreasing activity trends. However, significant improvements in anthropometric parameters, including body mass and waist circumference reduction, were observed independent of step-count changes. Quality of life improvements occurred without correlation to physical activity levels, suggesting that structured exercise interventions may provide therapeutic benefits through mechanisms beyond simple activity volume increases.

Our preliminary findings indicate that supervised Nordic Walking sessions may effectively improve specific health outcomes in individuals with schizophrenia through enhanced exercise quality, social engagement, and structured routine implementation rather than increased overall daily activity. These results suggest that structured group Nordic Walking sessions, adapted to patients’ psychophysical conditions and implemented continuously, represent a warranted recommendation for supporting treatment processes in psychiatric hospital settings. The intervention demonstrates potential as an adjunctive therapeutic approach that may enhance overall treatment outcomes while addressing the complex needs of hospitalized patients with schizophrenia, even when traditional activity metrics do not show improvement.

## Figures and Tables

**Table 1:** Shapiro–Wilk normality test summary.

Variable	W	*p*-Value	Distribution
BMI (Body Mass Index)	0.972	0.650	Normal
Waist Circumference	0.945	0.048	Non-normal
Hip Circumference	0.987	0.710	Normal
Weekly Step Counts	0.910	0.020	Non-normal
WHOQOL_pre (Quality of Life pre-intervention)	0.980	0.420	Normal
WHOQOL_post (Quality of Life post-intervention)	0.896	0.030	Non-normal
Body Mass	0.950	0.055	Normal
BMI Change	0.940	0.043	Non-normal
Waist Circumference Change	0.960	0.085	Normal
Hip Circumference Change	0.963	0.072	Normal
Calf Right	0.900	0.015	Non-normal
Calf Left	0.925	0.028	Non-normal
Plurimeter Thoracic Angle	0.980	0.340	Normal
Plurimeter Lumbar Angle	0.985	0.400	Normal
Chest Angle	0.970	0.210	Normal
Lumbar Angle	0.965	0.190	Normal

**Table 2:** Individual step-count trajectories with Holm correction.

Participant ID	Slope (Steps/Week)	R-Value	Raw p	p_adj (Holm)	Trend Direction
PFRON 01	−889.7	−0.463	0.2955	>0.05	Non-significant Decrease
PFRON 02	−277.5	−0.190	0.6825	>0.05	Non-significant Decrease
PFRON 03	−350.4	−0.623	0.1354	>0.05	Non-significant Decrease
PFRON 04	−10.1	−0.047	0.9198	>0.05	Non-significant Decrease
PFRON 05	238.1	0.367	0.4183	>0.05	Non-significant Increase
PFRON 06	897.9	0.593	0.2152	>0.05	Non-significant Increase
PFRON 07	325.2	0.809	0.0511	>0.05	Non-significant Increase
PFRON 08	479.1	0.379	0.4583	>0.05	Non-significant Increase
PFRON 09	−453.1	−0.308	0.5520	>0.05	Non-significant Decrease
PFRON 10	−66.0	−0.071	0.8942	>0.05	Non-significant Decrease
PFRON 11	−158.5	−0.349	0.5648	>0.05	Non-significant Decrease
PFRON 12	−478.7	−0.390	0.5163	>0.05	Non-significant Decrease
PFRON 13	−713.7	−0.799	0.0312	0.046	Exploratory Decrease (raw *p* < 0.01, p_adj > 0.05)
PFRON 14	−51.8	−0.105	0.8234	>0.05	Non-significant Decrease
PFRON 15	−17.7	−0.052	0.9119	>0.05	Non-significant Decrease
PFRON 16	40.8	0.216	0.6415	>0.05	Non-significant Increase
PFRON 17	−280.6	−0.990	0.0013	0.023	Exploratory Decrease (raw *p* < 0.01, p_adj > 0.05)
PFRON 18	−103.7	−0.187	0.6887	>0.05	Non-significant Decrease

**Table 3:** Individual WHOQOL pre/post scores, change, and cumulative steps.

Participant ID	WHOQOL_Pre	WHOQOL_Post	WHOQOL_Change	Total_Steps
PFRON 01	102	106	4	314
PFRON 02	65	69	4	314
PFRON 03	84	78	−6	317
PFRON 04	97	94	−3	293
PFRON 05	118	122	4	306
PFRON 06	52	55	3	250
PFRON 07	32	32	0	200
PFRON 08	50	62	12	280
PFRON 09	64	52	−12	270
PFRON 10	50	50	0	260
PFRON 11	30	33	3	230
PFRON 12	66	66	0	240
PFRON 13	68	69	1	250
PFRON 14	40	40	0	210
PFRON 15	1	1	0	220
PFRON 16	53	53	0	230
PFRON 17	40	40	0	240
PFRON 18	46	50	4	250
Mean (SD)	89.2 (15.7)	91.1 (15.5)	1.9(5.4)	-

**Table 4:** Results of paired *t*-tests and Wilcoxon signed-rank tests for pre- vs. post-intervention group means.

Parameter	t-Stat	Raw p (t)	Raw p (Wilcoxon)	p_adj (Holm)
Body Mass	−2.12	0.048	0.054	0.092
BMI	−2.05	0.053	0.062	>0.10
WHR	−1.19	0.253	0.188	>0.10
Waist Circumference	−2.45	0.027	0.042	0.081
Hip Circumference	−1.88	0.073	0.083	>0.10
Calf Right	0.55	0.591	0.736	>0.10
Calf Left	0.89	0.389	0.602	>0.10
Plurimeter Thoracic	−0.62	0.544	0.228	>0.10
Plurimeter Lumbar	−0.31	0.759	0.304	>0.10
Chest Angle	0.19	0.853	0.812	>0.10
Lumbar Angle	0.22	0.829	0.784	>0.10

p_adj: Holm–Bonferroni adjusted two-tailed *p*-value.

## Data Availability

The data presented in this study are available on request from the corresponding author, as they contain information that could compromise the privacy of research participants.

## Abbreviations

The following abbreviations are used in this manuscript:

AAF-NW	Adapted Activity Form—Nordic Walking
APA	Adapted Physical Activity
BMI	Body Mass Index
EPA	European Psychiatric Association
PA	Physical Activity
PFRON	State Fund for Rehabilitation of Persons with Disabilities
QoL	Quality of Life
WHOQOL-BREF World	Health Organization Quality of Life—BREF question
